# EXISTING DATA SETS FOR FAMILY CAREGIVING AND BEREAVEMENT RESEARCH: HIGH-VALUE APPLICATIONS AND KEY CONSIDERATIONS

**DOI:** 10.1093/geroni/igac059.1413

**Published:** 2022-12-20

**Authors:** Djin Tay, Rebecca Goodwin

## Abstract

Aging and the end of life is marked by greater reliance on others to address increasingly complex care needs. At the end of life, nine in 10 caregivers are family members. Reliance on family caregivers will continue to increase as the home outpaces hospitals and nursing facilities as place of death. As the population ages and dependence on family caregivers increases, it is critical to examine the impact of family on aging and end-of-life experiences and conversely, the impact of end-of-life caregiving and bereavement on families’ health and well-being. Population-based data, such as registries that link administrative, health and vital records or nationally representative datasets, minimize the challenges of selection bias, low response rates, and underrepresentation of racial and ethnic minority groups that are common in prospective clinical research. However, few population-based datasets exist that can facilitate research on family caregiving. This symposium includes five presentations focused on the use of population-based data for family caregiving research-- the first examines the social and caregiving network of adults who age solo using the National Health and Aging Trends Study data. The next two presentations examine how family characteristics are associated with place of death and end of life treatment utilization among nursing home decedents using linked statewide data. The final two examine how family caregivers are impacted by different death and dying experiences in Utah and Denmark. Leveraging population-based data to understand the end-of-life dynamics within a family system has the potential to advance family caregiving research, clinical practice, and policy.

